# Resting-state functional magnetic resonance imaging of the subthalamic microlesion and stimulation effects in Parkinson's disease: Indications of a principal role of the brainstem

**DOI:** 10.1016/j.nicl.2015.08.008

**Published:** 2015-08-21

**Authors:** Štefan Holiga, Karsten Mueller, Harald E. Möller, Dušan Urgošík, Evžen Růžička, Matthias L. Schroeter, Robert Jech

**Affiliations:** a Max Planck Institute for Human Cognitive and Brain Sciences, Stephanstr. 1A, Leipzig 04103, Germany; b Leipzig Research Center for Civilization Diseases & Clinic for Cognitive Neurology, University of Leipzig, Liebigstr. 16, Leipzig 04103, Germany; c Department of Stereotactic and Radiation Neurosurgery, Na Homolce Hospital, Roentgenova 2, Prague 15030, Czech Republic; d Department of Neurology and Center of Clinical Neuroscience, First Faculty of Medicine, Charles University In Prague, Kateřinská 30, Prague 12821, Czech Republic

**Keywords:** Parkinson's disease, Microlesion effect, Brainstem, Resting-state fMRI, Deep-brain stimulation, Subthalamic nucleus, BOLD, blood-oxygenation-level dependent, DBS, deep-brain stimulation, EC, eigenvector centrality, FDR, false discovery rate, fMRI, functional magnetic resonance imaging, FDG-PET, fluorodeoxyglucose positron emission tomography, FWE, family-wise error, GP, globus pallidus, ICA, independent component analysis, MLE, microlesion effect, MNI, Montreal Neurological Institute, PD, Parkinson's disease, PPN, pedunculopontine nucleus, rm-ANOVA, repeated measures analysis of variance, rs-fMRI, resting-state fMRI, SD, standard deviation, STN, subthalamic nucleus, UPDRS-III, motor part of the Unified Parkinson's Disease Rating Scale.

## Abstract

During implantation of deep-brain stimulation (DBS) electrodes in the target structure, neurosurgeons and neurologists commonly observe a “microlesion effect” (MLE), which occurs well before initiating subthalamic DBS. This phenomenon typically leads to a transitory improvement of motor symptoms of patients suffering from Parkinson's disease (PD). Mechanisms behind MLE remain poorly understood. In this work, we exploited the notion of ranking to assess spontaneous brain activity in PD patients examined by resting-state functional magnetic resonance imaging in response to penetration of DBS electrodes in the subthalamic nucleus. In particular, we employed a hypothesis-free method, eigenvector centrality (EC), to reveal motor-communication-hubs of the highest rank and their reorganization following the surgery; providing a unique opportunity to evaluate the direct impact of disrupting the PD motor circuitry *in vivo* without prior assumptions. Penetration of electrodes was associated with increased EC of functional connectivity in the brainstem. Changes in connectivity were quantitatively related to motor improvement, which further emphasizes the clinical importance of the functional integrity of the brainstem. Surprisingly, MLE and DBS were associated with anatomically different EC maps despite their similar clinical benefit on motor functions. The DBS solely caused an increase in connectivity of the left premotor region suggesting separate pathophysiological mechanisms of both interventions. While the DBS acts at the cortical level suggesting compensatory activation of less affected motor regions, the MLE affects more fundamental circuitry as the dysfunctional brainstem predominates in the beginning of PD. These findings invigorate the overlooked brainstem perspective in the understanding of PD and support the current trend towards its early diagnosis.

## Highlights

• DBS surgery in Parkinson's patients is often associated with a “microlesion effect” (MLE).
• Mechanisms behind MLE remain poorly understood.
• Using resting-state fMRI, we identified the brainstem as the principal hub responding to MLE.
• This invigorates the overlooked brainstem perspective in the understanding of Parkinson's disease.

## 1 Introduction

Deep-brain stimulation (DBS) is a rapidly evolving surgical strategy, in which one or more electrodes are implanted in specific brain regions to treat a variety of disabling neurological and psychiatric conditions. Externally-generated electrical currents applied to the electrodes then stimulate the surrounding brain tissue and eventually alleviate the patients' debilitating symptoms. While new applications and brain targets for DBS continue to emerge (Hariz et al., 2013), DBS of the subthalamic nucleus (STN) or globus pallidus (GP) interna have – over the past two decades (Miocinovic et al., 2013) – become well-established treatment options for movement symptoms associated with Parkinson's disease (PD).

Neurosurgeons and neurologists frequently observe an intriguing phenomenon, already in the operating room while implanting the DBS electrodes. Shortly after insertion of electrodes into the target structure, and well before the actual DBS pulse-generator is switched on, motor symptoms of many PD patients improve markedly. Such improvement remains noticeable for a certain period after implantation (Derrey et al., 2010; Koop et al., 2006; Singh et al., 2012), in some cases even months (Kondziolka and Lee, 2004; Mann et al., 2009). A general term, known as the “microlesion effect” (MLE), has been established to designate this phenomenon. Based on prevailing schemes of functional organization of the basal ganglia (DeLong, 1990), the MLE could be attributed to the reduction of abnormal basal ganglia output by disruption of cells and/or fibers in the STN and GP, that is, a mechanism similar to targeted ablative lesioning therapy. In fact, pallidotomy and subthalamotomy have been considered favored therapeutic approaches for patients with advanced PD in countries incapable of providing DBS treatment due to economical or technological limitations (Alvarez et al., 2005). However, in contrast to the permanent and irreversible impact of ablative surgery, DBS is considered a less destructive and more adaptable method (Haberler et al., 2000). Above all, the action of the MLE associated with implantation of the DBS electrodes is transient and gradually fades within days or weeks. This suggests that apart from the destruction of brain tissue within the electrode track, it reflects other transient posttraumatic tissue reaction along or close to the electrode. In particular, sharp leakage of neurotransmitters caused by damaged synapses influencing the surrounding unaffected neurons and post-operative collateral edema of brain matter around the electrode is thought to play an important role in the MLE (Jech et al., 2012). Appearance of the MLE is remarkably beneficial — is considered as an evident immediate sign of good placement of the DBS electrode within the particular portion of the target structure (Maltete et al., 2008). Yet, the mechanisms behind the MLE have not been understood.

We hypothesized that penetration of DBS electrodes in the STN has a substantial impact on the low-frequency blood-oxygenation-level dependent (BOLD) fluctuations in the resting-state (rs)-fMRI signal, which have been shown to be altered by PD (Jech et al., 2013; Kahan et al., 2014; Mueller et al., 2013). Specifically, the invasive intervention in combination with the utilization of a novel hypothesis-free analytical method gave us a unique opportunity to assess the direct impact of the physically disrupted STN on the rest of the abnormal motor circuitry *in vivo* without prior assumptions. This approach could identify novel anatomo-functional correlations accountable for improvement of motor symptoms related to the MLE and, subsequently, provide new insights into the functional organization of the human brain affected by PD.

## 2 Materials and methods

### 2.1 Subjects

Thirteen patients suffering from an akinetic-rigid variant of PD (11 males/2 females, age: 52 ± 7 years (mean ± standard deviation), disease duration since the first symptoms: 13 ± 3 years, duration of levodopa treatment: 9 ± 3 years) participated in the study after giving their written informed consent. All patients had a sporadic type of disease including five patients with beginning of symptoms before 40 years of age. None of these young-onset PD patients was positive for common genetic mutations potentially related to PD. All experimental procedures conformed to the Declaration of Helsinki, and the study protocol was approved by the Ethics Committee of the General University Hospital in Prague, Czech Republic. All patients met the United Kingdom Brain Bank Criteria for diagnosis of PD and were classified into the akinetic-rigid subtype of PD (Schiess et al., 2000), and suffered from motor fluctuations and/or disabling dyskinesias associated with long-term levodopa treatment. Further, all patients were referred for STN DBS therapy and thereby underwent the implantation of both DBS electrodes and the internal pulse generator.

Patients expressing signs of dementia and/or depression based on a standard psychiatric examination and neuropsychological testing (Mini-mental State Examination, Mattis Dementia Rating, Beck Depression Inventory) were excluded from the study. One further patient was excluded due to excessive involuntary movements extending to the head during the scanning session, which was evaluated post-hoc by analyzing realignment parameters of the data during the fMRI pre-processing procedure. A detailed demographic and clinical description of patients involved in the study is summarized in Table 1 and Supplementary Table S1.

### 2.2 Surgical procedure

Implantation of the DBS apparatus consisted of two separate surgery sessions: Insertion of the permanent quadripolar stimulation electrodes (3386 Medtronic, Minneapolis, MN, USA) into the STN bilaterally and implantation of the connection leads and the internal pulse generator to the subclavical region. This study focused exclusively on the time points preceding and immediately succeeding the first surgery session (*i.e.*, pre-/post-implantation of electrodes), while the MLE was still identifiable.

The methodology associated with implanting the DBS electrodes and the internal pulse generator was identical to the one thoroughly described elsewhere (Jech et al., 2012).

### 2.3 Magnetic resonance imaging

**Fig. 1:**
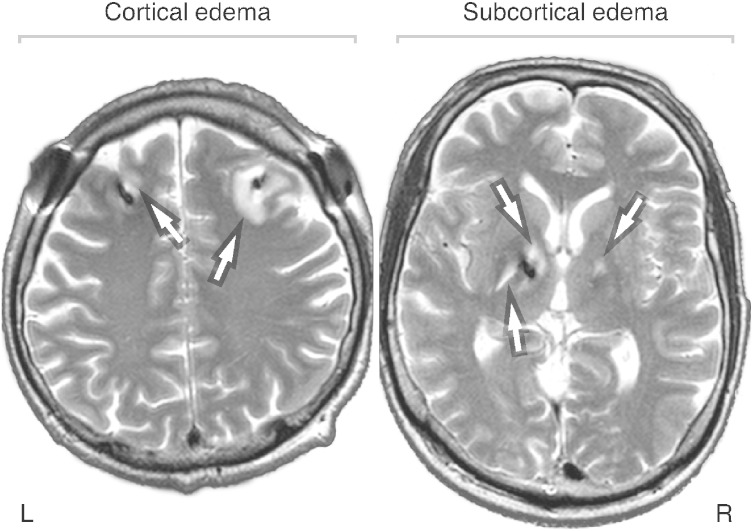
*T*_*2*_-weighted MR images demonstrating collateral edema caused by the DBS electrode insertion at the cortical (left panel; rating 5; patient 7) and subcortical levels (right panel; rating 3.5; patient 8). The edema appears as hyperintensity (indicated by arrows), while the DBS electrode artifacts are hypointense. MR — magnetic resonance; DBS — deep-brain stimulation; L — left and R — right.

All MRI investigations were performed at 1.5 T on a Magnetom Symphony scanner (Siemens, Erlangen, Germany). fMRI was collected in a 10-min session with 200 volumes of functional brain images collected using a T_2_*-weighted, gradient-echo-planar imaging (EPI) sequence (flip angle, *FA* = 90°; repetition time, *TR* = 3000 ms; echo time, *TE* = 51 ms, 31 axial slices, nominal in-plane resolution 3 × 3 mm^2^, slice thickness at 4 mm) sensitive to the BOLD effect. For display and registration purposes, high-resolution three-dimensional *T_1_*-weighted structural data were acquired using a magnetization-prepared rapid acquisition gradient-echo (MP-RAGE) sequence (*FA* = 15°; *TR* = 2140 ms; inversion time, *TI* = 1100 ms; *TE* = 3.93 ms). In Session 2 (*i.e.*, 0–3 days post-implantation), *T*_*2*_-weighted images were additionally collected using a turbo spin-echo sequence (*TR* = 5520 ms; *TE* = 86 ms; slice thickness 4 mm) for evaluating the grade of the collateral edema caused by the DBS electrode penetration. This particular imaging sequence markedly intensifies the contrast of edema (appearing as hyperintensities in resulting images; Fig. 1), which considerably eases its detection and evaluation.

All safety risks related to potential interference of the static magnetic field, radio-frequency pulses, or magnetic field gradient pulses and the implanted metallic DBS hardware were rigorously assessed, and associated technical precautions were diligently adhered to. The underlying safety standards employed for this evaluation have been published in more detail elsewhere (Jech et al., 2012).

### 2.4 Experimental protocol

Patients were instructed to look at a fixation cross on a projector screen while remaining still in a supine position during scanning. Two MRI examinations were carried out: (*DBS OFF1*) pre-implantation of DBS electrodes (‘Session 1’; 18 ± 17 days before implantation) and (*DBS OFF2*) post-implantation of DBS electrodes (‘Session 2’; 1 patient scanned on the day of surgery, 11 patients one day after surgery, and 1 patient 3 days after surgery). In order to uncouple the effect of electrode penetration from the therapeutic effects of levodopa and DBS, anti-Parkinsonian medication was withdrawn at least 12 h before both measurement sessions and the chronic DBS was not yet initialized. In both sessions (pre- and post-implantation), resting-state fMRI data sets (‘*DBS OFF1*’ and ‘*DBS OFF2*’, respectively) were acquired as described in the previous section. Additionally, the acute effects of DBS were tested at the end of ‘Session 2’ through the implanted electrodes using the identical rs-fMRI protocol (‘*DBS ON*’). A Dual Screen 3628 external stimulator (Medtronic, Minneapolis, MN, USA) was kept outside the MR scanner and connected to externalized leads of the electrodes with a 4-m extension cable that was used for DBS in a bipolar setup with parameters eliciting clear clinical improvement (mean amplitude: 2.64 ± 0.44 V, pulse duration: 60 µs, pulse frequency 130 Hz). Due to technical reasons, two separate rs-fMRI acquisitions were made during unilateral left and unilateral right neurostimulation (in random order) each lasting up to 15 min.

### 2.5 Evaluation of motor symptoms

Severity of patients' PD motor symptoms was assessed off medication using the UPDRS-III score by a movement disorder specialist (R.J.) prior to every rs-fMRI measurement (Sessions 1 and 2). The UPDRS-III score off medication was also evaluated in two additional post-operative sessions without fMRI scanning: ‘Session 3’ approximately 1 month after surgery, immediately preceding the initiation of chronic DBS treatment by the internal pulse generator at optimal settings with DBS both off and on; and ‘Session 4’ (with the exception of one patient) at a later post-operative phase 1 year after implantation during active DBS when optimal stimulation parameters were reached. *T*_*2*_-weighted images were also collected during Session 4 at the latter post-operative stage 1 year after implantation for edema assessment (without the fMRI measurement).

The UPDRS-III score and the hemibody, axial, akinesia, rigidity, and tremor subscores were systematically extracted from the UPDRS-III score sheets for more elaborate, symptom-driven fMRI analyses (Holiga et al., 2013) (see the fMRI analyses section). To assess changes in patients' motor symptoms severity pre- and post-implantation, the UPDRS-III score was evaluated using a repeated measures analysis of variance (rm-ANOVA). Prior to rm-ANOVA statistics, the Shapiro–Wilk test of normality and Mauchly's test of sphericity were performed to verify the assumptions of normality and sphericity in the distribution of data. *Post-hoc* analysis was performed in order to reveal significant UPDRS-III changes between particular measurement sessions (*i.e.*, pre-implantation, 0–3 days post-implantation, 1 month post-implantation, 1 year post-implantation).

### 2.6 Edema assessment

The grade of collateral edema associated with penetration of the electrodes was evaluated individually by an average semi-quantitative rating of two blinded raters in both cortical (affecting the white matter just beyond the cortex) and subcortical regions (affecting deep structures and adjacent white matter) for each hemisphere separately. This evaluation was performed by assessing *T*_*2*_-weighted MR images acquired during Session 2 (Fig. 1). We adopted the rating scale from our previous work (Jech et al., 2012). Briefly, the higher the rating, the larger the area of detected cortical/subcortical edema (range 0–6 for each), with 0 representing no collateral edema around the electrodes.

#### 2.6.1 fMRI analyses

The data were pre-processed using the statistical parametric mapping (SPM8) software package patched to revision 4667 (Wellcome Trust Centre for Neuroimaging, UCL, London, UK), Matlab (R2010b, MathWorks Inc., Natick, MA, USA) and Lipsia software package (Lohmann et al., 2001). A standard pre-processing of fMRI data was performed including realignment, slice-time correction, normalization to the Montreal Neurological Institute (MNI) template, spatial filtering using an 8-mm full-width-at-half-maximum three-dimensional Gaussian kernel, and a temporal filtering using a band-pass filter passing the frequencies within a range of 0.0125–0.2 Hz.

The motion parameters obtained from the SPM realignment procedure (three translational; three rotational) were further investigated for differences of motion in the pre- and the post-implantation states.

#### 2.6.2 Eigenvector centrality (EC) mapping

In the current work, we assessed the effect of the STN microlesion on PD brain networks by means of functional connectivity, that is, correlations of BOLD signal fluctuations between distal brain regions over the course of an rs-fMRI session recorded in the absence of experimental stimulation or task (Fox and Raichle, 2007; Zhang and Raichle, 2010). The inability to extract the MRI information from the DBS target region itself – due to magnetic susceptibility artifacts caused by the implanted metallic electrodes – disallowed us to assess the connectivity of the disrupted STN directly. Therefore, in contrast to conventional hypothesis-driven rs-fMRI experiments, where a seed region of interest is specified *a priori* (Buckner et al., 2013), we employed EC (Lohmann et al., 2010) as an assumption-free, hypothesis-independent exploratory method based on graph theory (Bullmore and Sporns, 2009, 2012).

Thesis of EC was already established at the beginning of the 20th century and has been continuously exploited in various contexts ever since (*e.g.*, in the context of ranking webpages, scientific journals, or economic sectors) (Franceschet, 2011). By using EC mapping (Lohmann et al., 2010), these iterative constructs are extrapolated into the world of functional connectivity and rs-fMRI. Subsequently, we investigated spatial reorganization of *central* brain regions – in the current case, motor communication hubs – following the penetration of the DBS electrodes and subsequent stimulation in the STNs of 13 patients suffering from advanced PD.

**Fig. 2:**
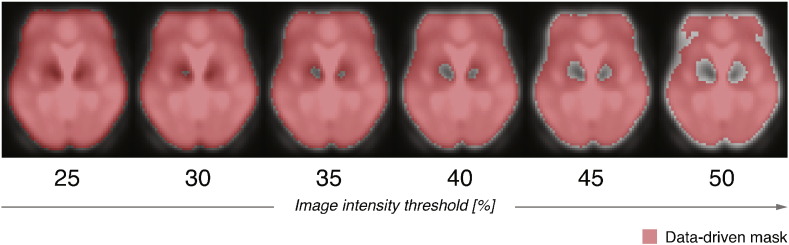
Data-driven masking procedure based on image intensity thresholding to account for magnetic susceptibility artifacts caused by the metallic DBS electrodes and the pulse generator. Various image intensity thresholds ranging from 0.25 to 0.50 were used for qualitative assessment of the artifacts and formation of the search space for subsequent ECM calculations. The masks are overlaid on an average normalized fMRI obtained in the post-surgery sessions. DBS — deep-brain stimulation and ECM — eigenvector centrality mapping.

**Fig. 3:**
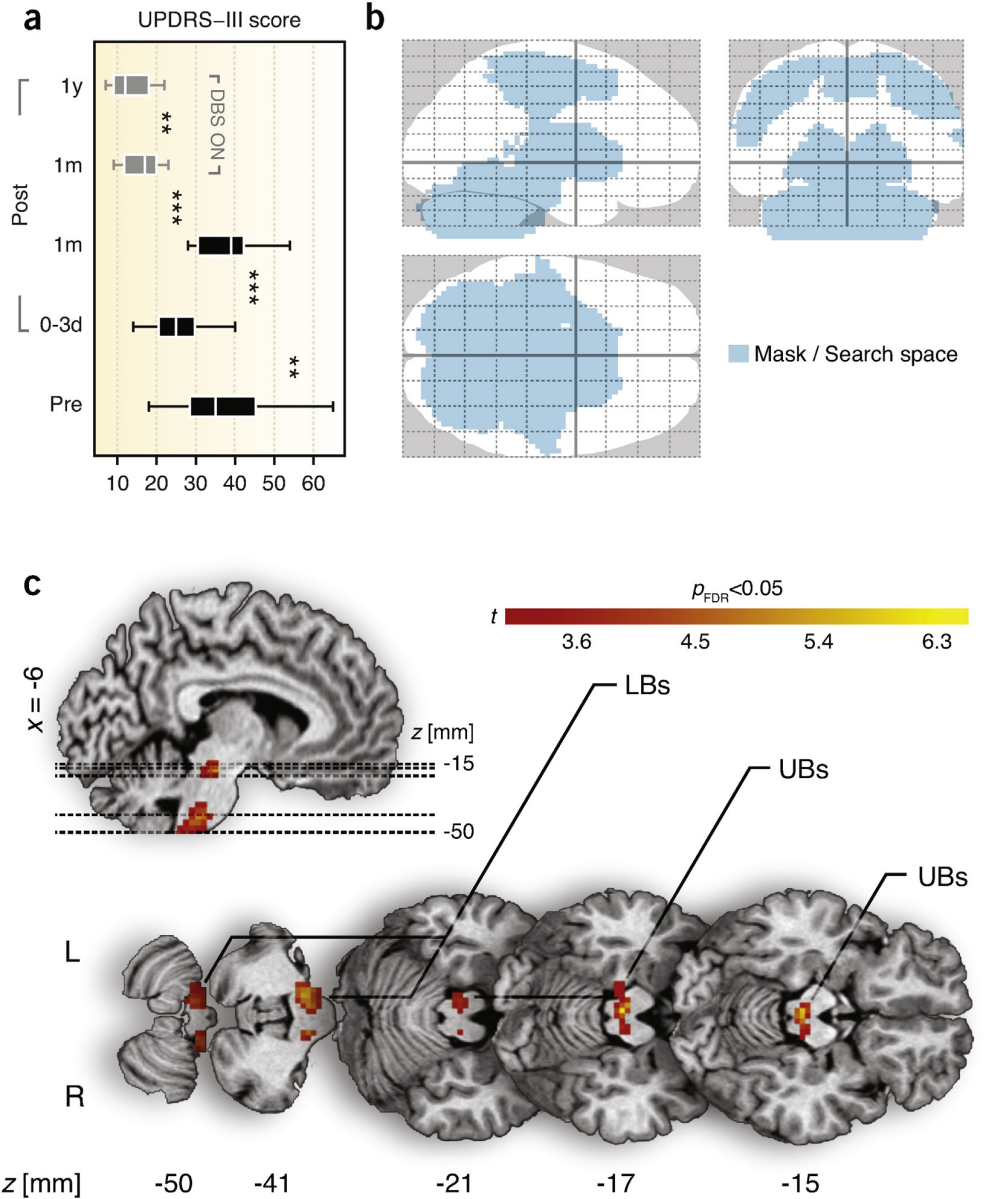
Impact of DBS electrode implantation in the STN on motor networks of 13 patients suffering from PD in the absence of medication and with DBS switched off. (a) UPDRS-III scores indicating alleviation of PD motor symptoms in the acute phase of microlesion followed by a relapse to the pre-operative levels in the latter stages after implantation. In addition, effect of STN DBS is shown in gray bars 1 month and 1 year after implantation. The bars show mean, first/third quartile, and lower/upper adjacent values. (b) Outlined search space (region-of-interest) used for EC calculations and subsequent statistics comprising the entire motor system of the brain overlaid on a standardized stereotactic brain. (c) Reorganization of *central* motor communication hubs due to microlesion effect following the DBS electrode penetration in the STN. The brainstem was identified as the *central* functional connectivity hub sensitive to microlesion. Summary of obtained statistics is shown in Table 2. 0–3 d — days 0–3; 1 m — 1 month; 1 year — 1 year; DBS — deep-brain stimulation; EC — eigenvector centrality; FDR — false discovery rate; L — left; LBs — lower brainstem; MLE — microlesion effect; PD — Parkinson's disease; Post — post-implantation stage(s); Pre — pre-implantation stage; R — right; STN — subthalamic nucleus; UBs — upper brainstem; and UPDRS-III — motor part of the Unified Parkinson's Disease Rating Scale; ** — *p* < 0.01; *** — *p* < 0.001.

The foundation of EC is a similarity matrix (encompassing a measure of goodness of some kind) — in our case an *n* × *n* symmetric correlation matrix, **X** (where *n* represents the number of voxels). The entries of this matrix denote correlations between fMRI timecourses of a particular pair of voxels. In mathematical terms, EC of a voxel *x_i_* is defined as the *i*th entry in the normalized eigenvector belonging to the largest eigenvalue of **X**. To ensure the uniqueness and non-negativity of the largest eigenvalue and associated eigenvector components by the Perron–Frobenius theorem, the correlation matrices were rescaled prior to EC calculations by adding one to each element of the matrix. We refer the reader to the publication by Lohmann et al. (2010) for a more formal and detailed description of EC mapping for fMRI. Using the Lipsia software package (Lohmann et al., 2001), we calculated the individual eigenvectors (EC maps) for each patient and measurement session separately (*i.e.*, Sessions 1 and 2, pre-/post-implantation of electrodes). In other words, we used the iterative character of EC to identify the most *central* communication hubs – regions functionally connected with many other *central* regions – in the motor system of each patient pre- and post-surgery. Importantly, the EC calculations were restricted to regions masked in a search space comprising the motor system specifically (Fig. 3b; premotor, motor and sensory cortices, basal ganglia, brainstem, and cerebellum) based on a WFU PickAtlas standard human brain atlas (Maldjian et al., 2003), to restrain EC contributions of areas of no interest presumably affected by the experimental paradigm (*e.g.*, visual system). In addition, fMRI voxels exhibiting severe magnetic susceptibility artifacts caused by the presence of the DBS apparatus in the static magnetic field were excluded from the search space used in all subsequent analyses and statistics. In particular, a qualitative approach was adopted based on thresholding of individual post-surgery, distorted fMRI data. Various intensity cutoff-thresholds ranging from 0.25 (liberal) to 0.50 (conservative) with a step size of 0.1 were used to generate a group, data-driven mask for EC calculations. Fig. 2 demonstrates the approach by depicting various intensity thresholds that were employed for the qualitative assessment of the resulting masks.

Clearly, liberal thresholding (0.25) results in a mask incorporating regions around the STN affected by electrode-induced artifacts. In contrast, a threshold of 50% is overly conservative and eliminates a considerable portion of artifact-free brain areas. After visual inspection, a moderately conservative threshold of 45% was chosen for creating the data-driven mask. The final mask was then formed as a conjunction between the anatomical search space and the intensity-thresholded mask. Of note, the resulting parametric maps remained identical regardless of the selected data-driven masking threshold, which suggests that the magnetic susceptibility artifacts did not affect the correlation patterns identified by ECM.

To reveal reorganization of the most important communication hubs related to the insertion of electrodes across the group of patients, a group analysis in the form of a paired *t*-test (EC maps post-implantation, off stimulation — EC maps pre-implantation) was performed. The effects of age, disease duration, and levodopa treatment duration were also investigated using additional covariates in the statistical model. Here, we computed the interaction between these factors and the effects of electrode penetration to exclude a different correlation between the covariates and EC in the pre- and post-implantation states. To disentangle the microlesion-related functional reorganization from actual DBS effects, we also computed a paired *t*-test between the *DBS ON* and *DBS OFF2* state. An uncorrected voxel-level threshold of *p* < 0.005 was adopted with a *post-hoc* cluster-size adjustment to preserve only clusters corrected for multiple tests using the false discovery rate (FDR) correction at *p*_FDR_ < 0.05. This particular alpha level was used in all subsequent analyses.

A more conventional seed-based approach was also performed to demonstrate the “destination” regions driving the ECM changes caused by the MLE. The peak voxel identified by the paired *t*-test was used as a seed voxel. Correlations between timecourses of the seed region and the rest of the brain were calculated in pre- and post-surgery data. The correlation maps were transformed using Fisher *r*–*z* transformation and a paired *t*-test was calculated using a “post–pre” contrast.

To disentangle the microlesion-related reorganization of intrinsic activity from the actual DBS effects, we also performed a statistical analysis assessing potential differences between EC obtained within the post-implantation sessions. Here we used a flexible-factorial model containing the EC of all three measurements of the post-implantation session (*DBS OFF*, left *STN DBS ON*, right *STN DBS ON*). The final contrast was set to check for significant EC response to DBS.

Additionally, a linear regression group model was fitted to discover potential linear relationships between the EC maps and the particular UPDRS-III scores/sub-score (hemibody, axial, akinesia, rigidity, tremor) (Holiga et al., 2013), but also edema ratings, irrespective of the stage of surgery. A dot-plot was formed to illustrate the linear relationship between adjusted EC response at a specific brain coordinate and the UPDRS-III score for each patient/stage (13 patients × 2 stages). Thereupon, coefficient of determination (*R*^2^) was calculated to quantify the proportion of variance explained by the model.

## 3 Results

### 3.1 Clinical scores

The motor part of the Unified Parkinson's Disease Rating Scale (UPDRS-III) score differed substantially across the measured time points pre- and post-surgery (Fig. 3a). It dropped significantly (*p* = 0.009; *F*(1,12) = 9.5) from 34.2 ± 9.9 (mean ± standard deviation) in the first measurement session (3–59 days pre-surgery, off medication) to 25.8 ± 7.3 in the second measurement session (0–3 days post-surgery, off medication, off stimulation) evidencing the therapeutic benefit of the electrode penetration and, thereby, demonstrating the MLE itself. In the third measurement point (approx. 1 month after surgery, before the initiation of chronic DBS treatment), the UPDRS-III in absence of stimulation leveled up with the UPDRS-III in the pre-operative level, thus no significant difference could be found between them (*p* = 0.228). In particular, the score increased to 37.5 ± 7.7, indicating the transitory character of the MLE and its eventual deterioration over a period of 1 month. Immediately after initiation of the stimulation 1 month after implantation (off medication), the score improved to 16.5 ± 4.6 (*p* = 2.6 × 10^−4^; *F*(1,12) = 26.1 compared to the first session; *p* = 7.0 × 10^−6^; *F*(1,12) = 56.6 compared to the third session), documenting the success of the surgical intervention and the remarkable therapeutic benefit of DBS in the study group, even at the initial stages of chronic DBS treatment. In the fourth measurement session (approx. 1 year upon chronic DBS treatment), the UPDRS-III off medication decreased even further to 12.3 ± 4.2 (*p* = 0.014; *F*(1,11) = 8.5) in comparison to the measurement taken immediately after initiation of DBS (approx. 1 month after surgery), confirming the beneficial long-term character of the DBS treatment and correctness of electrode placement.

#### 3.1.1 Edema

Penetration of the DBS electrodes caused acute collateral edema along the track of the electrodes (Fig. 1). The edema was assessed in the first post-operative stage (0–3 days after surgery). Subcortical and cortical edemas were rated at 1.7 ± 1.5 and 2.8 ± 1.7, respectively (range 0–6). No significant left-/right-side edema differences were found in either cortical or subcortical structures. One month after implantation, the edema gradually vanished, as indicated by UPDRS-III scores, which returned to pre-operative levels (Fig. 3a). No signs of edema were detected 1 year post-implantation, suggesting that edema is a principal mechanism contributing to the beneficial effect of DBS electrode penetration.

#### 3.1.2 Motion inside the scanner

The analysis of the motion variables did not show a significant difference between the subject's motion in the pre- and the post-implantation state including the mean, the standard deviation, and the maximum of the six motion parameters (*p* > 0.05, uncorrected).

#### 3.1.3 Eigenvector centrality mapping

**Fig. 4:**
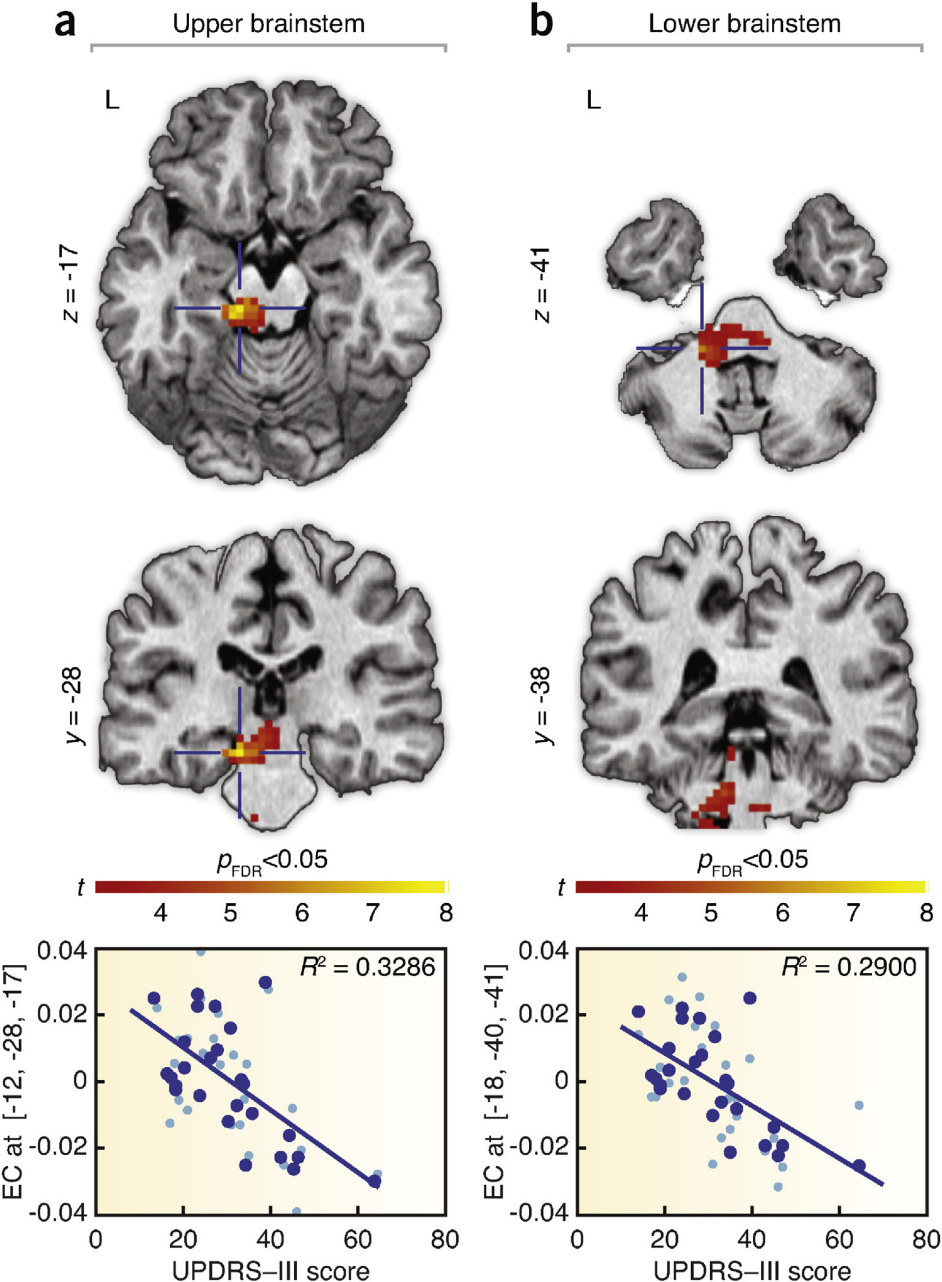
Statistical maps of the voxel-wise negative correlations between EC and motor symptoms of 13 patients regardless of the implantation stage (*i.e.*, the better the clinical picture of patients, the higher the EC in a particular brain region). UPDRS-III scores significantly inversely correlated with the EC of the (a) upper and (b) lower brainstem. Bottom graphs denote the particular linear relationship in a respective voxel coordinate indicated by a blue cross. EC responses are fitted, normalized, and displayed as blue dots. The light blue dots indicate the EC responses plus errors. EC — eigenvector centrality; FDR — false discovery rate; *R*^2^ — coefficient of determination; and UPDRS-III — motor part of the Unified Parkinson's Disease Rating Scale.

The contrast of potential microlesion effects (post-implantation, *DBS OFF2*–pre-implantation, *DBS OFF1*) showed a significant increase of EC due to electrode penetration in the upper and lower brainstem (Fig. 3c; Table 2); forming the main intrinsic communication hub responding to the penetration of electrodes. The result was not affected by including age, disease duration, and treatment duration as additional covariates. The interaction analysis between these covariates and the pre- and post-implantation state did not show any significant effect. The reversed contrast (*i.e.*, pre-implantation, *DBS OFF1*–post-implantation, *DBS OFF2*) did not reveal any significant EC reduction after implantation of electrodes. In addition, EC inversely correlated with UPDRS-III scores in the upper and lower brainstem, irrespective of the surgery stage (Fig. 4a and b). No relationship was found between the EC maps and the edema ratings.

**Fig. 5:**
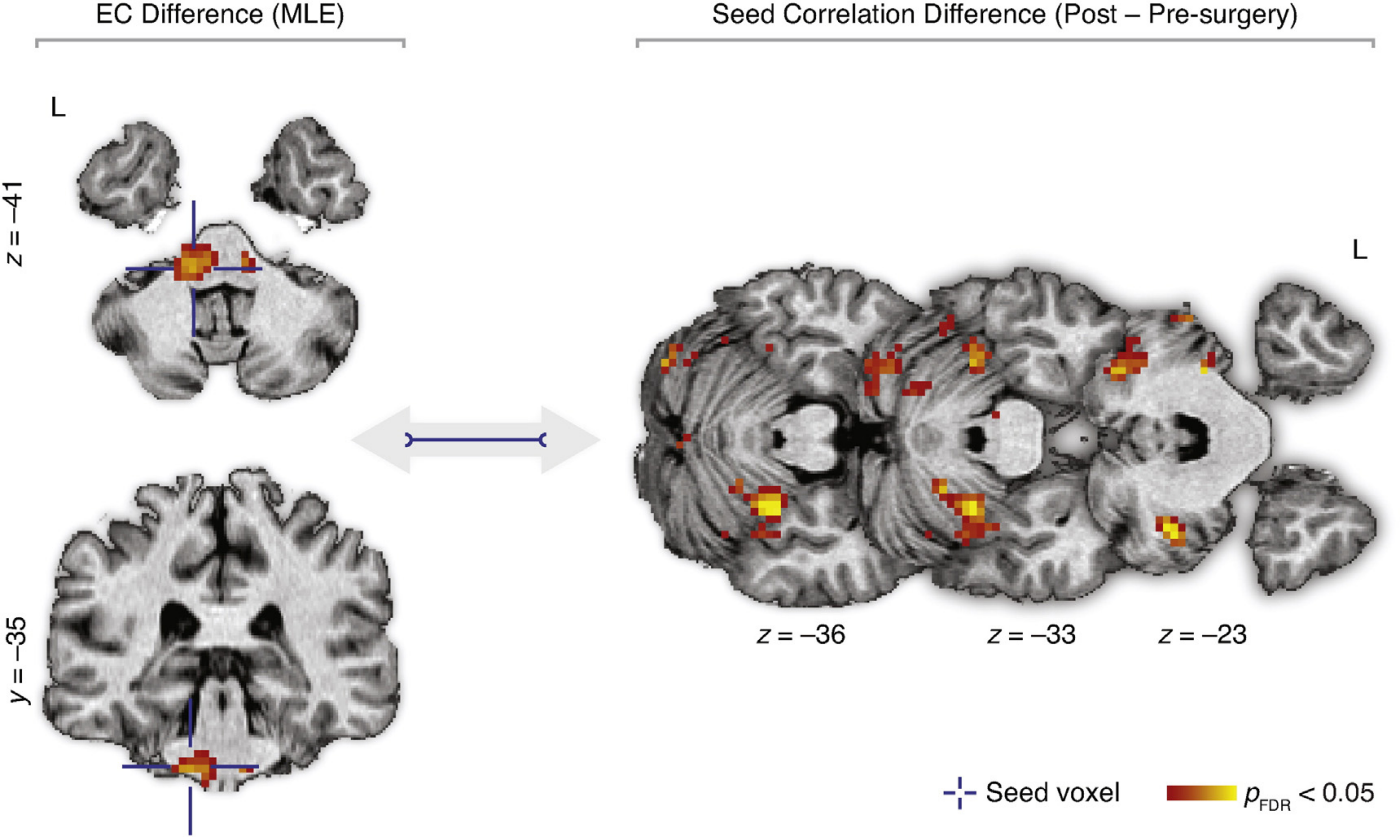
Seed-based correlation differences caused by penetration of the DBS electrodes in the STN. Insertion of electrodes caused increased synchronization between the brainstem and the cerebellum. EC — eigenvector centrality; FDR — false discovery rate; MLE—microlesion effect; STN—subthalamic nucleus; and UPDRS-III — motor part of the Unified Parkinson's Disease Rating Scale.

**Fig. 6:**
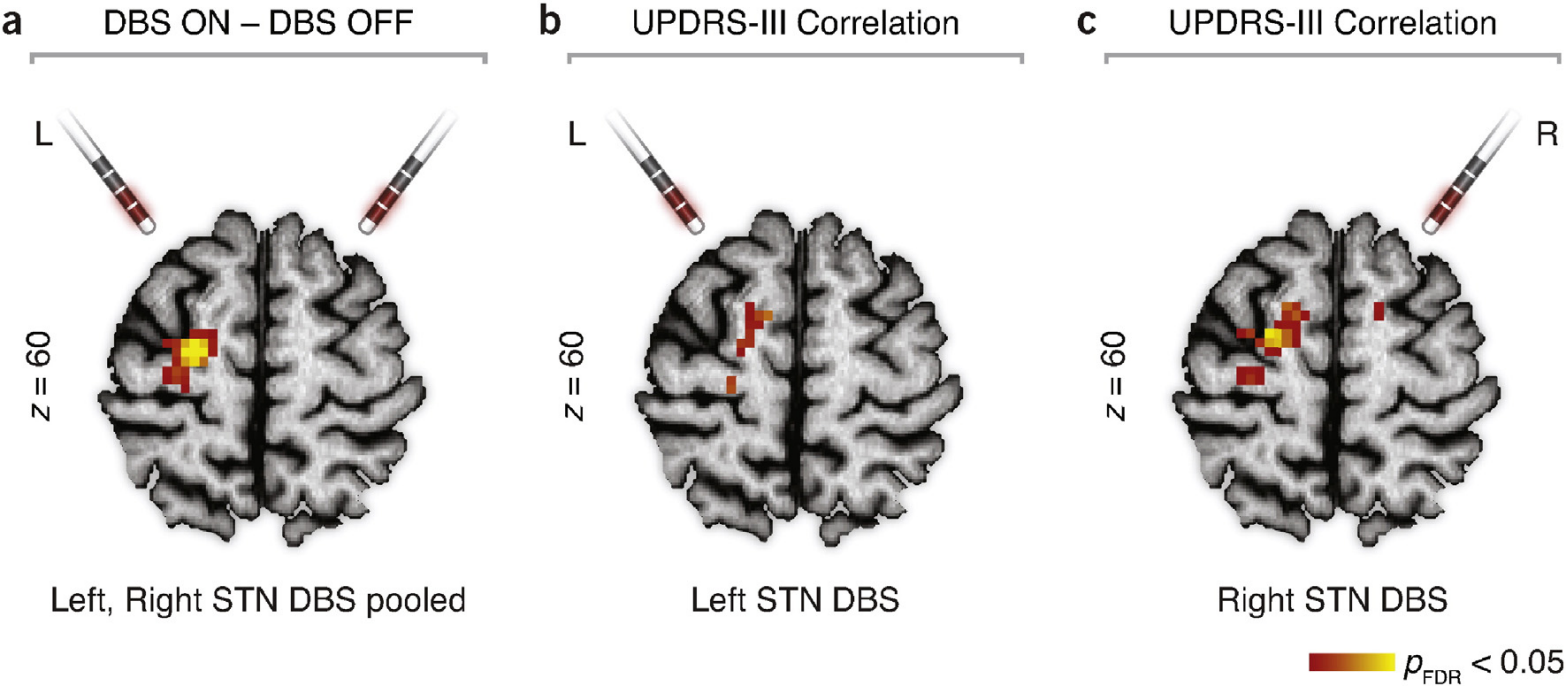
EC change related to acute DBS. (a) Treating the patients with unilateral STN DBS increased EC in the PMC. Data from left and right unilateral stimulation were pooled in the model. (b) Correlation of left STN DBS with the UPDRS-III scores. (c) Correlation of right STN DBS with the UPDRS-III scores. DBS — deep-brain stimulation; EC — eigenvector centrality; FDR—false discovery rate; STN — subthalamic nucleus; UPDRS-III — motor part of the Unified Parkinson's Disease Rating Scale.

Conventional seed-based approach revealed significantly increased synchronization between the brainstem peak and several cerebellar regions (Fig. 5). For the *DBS ON* (conjunction analysis from unilateral left and unilateral right neurostimulations) compared to the *DBS OFF2* state, there was an increase of EC in the left premotor cortex (Fig. 5). In addition, EC inversely correlated with UPDRS-III scores in the left premotor cortex, irrespective of the session (Fig. 6a, b and c).

## 4 Discussion

### 4.1 Study overview

In this study, we assessed the effects of DBS electrode penetration in brains of patients undergoing STN DBS surgery to determine how the invasive surgical procedure alone alters the function of motor circuitry in PD, and to identify the mechanisms responsible for the considerable motor improvement that patients experience in the acute phase of microlesion before initiation of DBS. The electrode insertion in the STN had an eminent impact on the intrinsic activity of the motor system as evaluated by correlations of spontaneous low-frequency BOLD fluctuations measured by rs-fMRI. The major finding was the identification of the brainstem as the principal hub with increased centrality of functional connectivity following the electrode penetration (Fig. 3c; Table 2), and as the region particularly recruited in the amelioration of the motor symptoms (Fig. 4a and b).

### 4.2 Role of the brainstem

Significant contribution of the brainstem in PD pathology was already established six decades ago (Eadie, 1963; Greenfield and Bosanquet, 1953) and was corroborated by a more recent diligent histopathological study of Braak et al. (2003) who recognized the importance of the brainstem pathology especially in the initial, prodromal phases of the disease (Snyder et al., 2013; Tison and Meissner, 2014). Strikingly, closed-loop subcortical projections linking the brainstem structures with the basal ganglia were proposed and regarded as phylogenetically older than the well-known cortical–basal ganglia–cortical connectional architecture (McHaffie et al., 2005), which might indicate the potentially paramount significance of the brainstem structures in the disease pathogenesis. A recent study has confirmed this proposal; specifically, in addition to ascending dopaminergic projections from substantia nigra pars compacta and/or ventral tegmental area to basal ganglia, descending projection to mesencephalic locomotor region – a brainstem region controlling locomotion – was discovered in lampreys (Ryczko et al., 2013). Also, dopaminergic innervation of pedunculopontine nucleus (PPN), another part of the brainstem (role of PPN in PD is reviewed in Pahapill and Lozano (2000)), was observed in monkeys with its radical reduction after intoxication with MPTP (1-methyl-4-phenyl-1,2,3,6-tetrahydropyridine) — a primate model of PD (Rolland et al., 2009). These and other (Grinberg et al., 2010) anatomo-pathological substrates for brainstem and basal ganglia interactions therefore represent a solid basis for the findings presented in the current study.

Recently, there have been radical changes in the current concepts of structural and functional roles of cerebellar circuits, by recognizing anatomical substrates for cerebellum influencing the basal ganglia and vice-versa, and by observing that cerebellar output is affecting generation and control of movement at the motor cortex levels (Bostan et al., 2013). A review by Wu and Hallett (2013) signifies the considerable involvement of cerebellum in PD. It is speculated to exert both pathological and compensatory effects, though the current knowledge about its role in PD pathophysiology remains rather limited (Wu and Hallett, 2013).

Our results suggest that the brainstem – by significantly inflating centrality of connectedness and increasing synchronization with the cerebellum – acts as a compensatory hub supporting the disrupted motor network to maintain relatively normal motor function in the acute phase of microlesion. The roles of the brainstem and the cerebellum, however, do not seem to be exclusively supportive and compensatory. Results from recent rs-fMRI studies investigating intrinsic brain activity of PD patients also clearly imply integration of the brainstem and the cerebellum in the disease pathophysiology and its treatment. In particular, excessive modulation of the brainstem's EC by levodopa was revealed in patients with PD (Jech et al., 2013). Also, reductions in the brainstem's functional connectivity with the striatum were identified in PD patients as compared to healthy subjects (Hacker et al., 2012). Seed-based analyses of striatal networks revealed increased functional connectivity of the cerebellum with putamen following the dopaminergic challenge (Kelly et al., 2009). Further, decreased striatal functional connectivity of cerebellum was observed in PD patients as compared to healthy subjects (Hacker et al., 2012) and pathological functional connectivity interactions between cerebellothalamic and basal ganglia circuits were also reported in tremor-dominant PD patients (Helmich et al., 2011). These findings indicate direct involvement of the brainstem and the cerebellum in degenerated dopaminergic networks and point to their significant contribution to PD-related pathological processes. This reinforces the relevance of our finding (Fig. 3c; Table 2) that the brainstem constitutes a central communication hub and, by increasing its functional connectivity with the cerebellum, particularly supports the physically-disrupted motor system during the acute phase of microlesion in PD. However, the brainstem also simultaneously renders pathological effects, as documented by the significant linear association of the motor symptoms of the disease with the brainstem's EC (Fig. 4a; Fig. 4b). Therefore, we speculate that the central role of the brainstem may include two overlaying perspectives — compensatory and pathological.

As mentioned earlier, Braak et al. (2003) observed that the neural damage does not spread randomly in the human brain affected by PD, but rather follows a predetermined spatio-temporal sequence within distinct stages, beginning in the lower brainstem — the medulla oblongata in the presymptomatic phase (stages 1–2), ascending to pontine tegmentum and midbrain, when the first motor symptoms appear (stages 3–4), and eventually converging in the neocortex with the emergence of non-motor symptoms (stages 5–6). This emphasizes the importance of the lower brainstem pathology in the disease progression, especially in its presymptomatic phases, when the magnitude of neural loss is still minimal, no motor symptoms are apparent, hence the chances of discovering putative disease-modifying therapies, which would eventually halt the disease progression, are higher (Goedert et al., 2013; Tison and Meissner, 2014). The brainstem regions therefore have the uttermost potential to deliver preclinical markers of the disease and are predisposed as the potential target for testing novel neuroprotective agents or brain stimulation techniques (Abbott, 2005; Pahapill and Lozano, 2000). Research attempts should therefore shift from the striatal dogma towards earlier, premotor phases of the disease and turn appropriate attention to the overshadowed brainstem. The work presented here demonstrates that the EC of intrinsic activity as measured by rs-fMRI correlations is capable of detecting changes even in the lower brainstem structures and thus might be a potential candidate for premotor biomarkers of the disease.

### 4.3 Previous imaging studies of the STN microlesion

Our previous work revealed significantly decreased amplitude of finger-tapping-related BOLD response in several areas including precentral gyrus, supplementary motor area, rolandic operculum, insula, thalamus, and GP/putamen following the implantation of electrodes in the STN (Jech et al., 2012). Although intriguing, comparing the current rs-fMRI and previous task-based fMRI observations does not provide much room for interpretation, as the relationship between intrinsic low-frequency BOLD fluctuations and evoked task-based BOLD activity is not entirely known (Fox et al., 2005; Smith et al., 2009).

Models of the relationship between functional connectivity and energy consumption have been, on the contrary, recognized recently (Tomasi et al., 2013), proposing a non-linear power scaling of the degree of connectivity and glucose metabolism in the human brain. Therefore, it is critical to note that no correspondence has been identified between presently observed EC changes and previously reported FDG-PET changes (Pourfar et al., 2009) related to the STN microlesion. It is important to point out the dissimilar experimental designs used within the studies, which could have caused the dichotomous observations. While in our study, brain data were acquired 0–3 days post-implantation from 13 DBS-naïve, akinetic-rigid PD patients, Pourfar et al. (2009) performed PET scanning at an average of 20 months post-operatively on 6 patients of unknown PD subtype who had already undergone chronic DBS treatment. Although Pourfar et al. controlled for the implantation-to-scanning-period variance in their statistical analyses, this might have been insufficient to account for confounds likely influencing the results, *e.g.*, the action of transient collateral edema, or the potential short-term impact of chronic DBS treatment introducing consequent after-effects in absence of stimulation.

### 4.4 Relation to DBS

Interestingly, an increase of EC in the premotor cortex was identified in the same group of patients in response to the STN DBS treatment bilaterally (Mueller et al., 2013), although an increase of EC was more pronounced in the left premotor cortex. Eigenvector centrality correlated with UPDRS-III scores, indicating clinical effects of the functional connectivity changes. These effects might be transmitted by the so-called hyperdirect pathway between STN and cortical motor areas as shown by connectivity analyses in healthy subjects (Brunenberg et al., 2012). Taken together, these data and our present results do not suggest an overlying intrinsic brain connectivity mechanism in MLE and DBS. Surprisingly, while DBS of STN seems to act (with regard to EC) at the cortical levels only, sole penetration of electrodes caused functional connectivity changes only in ventral posterior regions of the brain. It is noteworthy that the fMRI scans in both cases were taken in the acute phase of microlesion, before the chronic treatment with the DBS was initiated. It seems that the mechanisms behind acute microlesion did not directly contribute to the DBS response, as no significant interaction has been found between the EC responses of the two clinical interventions. Nevertheless, the results of Mueller et al. (2013) clearly reconcile with the simple model of STN inhibition leading to an increase in cortical activation (Ballanger et al., 2009).

### 4.5 Limitations of the study

EC mapping has a major advantage over other data-driven ontologies as independent component analysis (ICA). While ICA requires assumption on the number of components to be used, which can result in some complications (Zhang and Raichle, 2010), no *a priori* information is needed before EC is performed. This is likely to be advantageous in view of substantial distortion artifacts and signal voids in the fMRI data acquired with echo planar imaging (EPI) due to a strong gradient in the local magnetic susceptibility (and, hence, magnetic field inhomogeneity) in the vicinity of the DBS electrodes and the unknown effect of these artifacts on the known functional networks. However, voxels exhibiting very low signal (STN, premotor cortical areas) needed to be excluded from the EC mapping search space, together with a considerable portion of brain not assumed to be involved in motor aspects of PD pathophysiology. This might have resulted in missing certain centrality patterns relevant for PD. Specifically, excluding voxels of the disrupted, later stimulated, thus clearly the most essential part of the network – STN – possibly resulted in decreased sensitivity to detect hubs interconnected with it. Further, EC does not reveal the directionality of functional connectivity and is unable to uncover interactions between identified hubs, thus no causal description of intrinsic activity differences could be derived.

Low resolution of MR images acquired at 1.5 T consequently limited the inspection of potential mechanisms behind the MLE on a rather coarse spatial scale. More detailed analyses with much greater spatial accuracy are needed to attribute the action of the MLE to certain physiological processes (*i.e.*, confirming leakage of neurotransmitters or observing transitory mechanisms of edema). The insufficient resolution of the images also disallowed us to localize the centrality changes within the brainstem's internal architecture precisely.

Complicated surgical procedures and associated ethical constraints restricted us in randomizing the experimental design properly. Specifically, there was almost no variance in implantation-to-measurement time, which limited us in studying the edema spreading over time and observing its direct contribution to the MLE in more detail.

## 5 Conclusion

The invasive intervention in combination with the hypothesis-free analytical method of ECM provided us with a unique opportunity to observe the direct and cardinal impact of dysfunctional STN on the resting motor-circuitry in PD patients *in vivo*. Despite the similar benefit on motor functions, the transitory MLE and DBS were associated with anatomically different patterns of resting functional connectivity. While the DBS propagates to premotor areas suggesting compensatory activation of less affected cortical regions, the MLE affects the fundamental circuitry itself as the brainstem dysfunction predominates since the early stage of PD. The MLE-related activation of the brainstem and cerebellum presumably compensates for the disrupted neurons to maintain relatively normal motor function in the acute phase of MLE, but possibly exerts pathological effects, too. Our current findings thus support the necessity of increased emphasis on brainstem and cerebellum in the research of PD.

The following are the supplementary data related to this article.

Supplementary Table S1 **UPDRS-III subscores of the 13 patients included in the study. ID** — patient’s identification number; **s1OFF** — subscore in the first scanning session off medication (pre-implantation); **s1ON** — subscore in the first scanning session on medication (pre-implantation); **s2OFF** — subscore in the second scanning session off medication and off stimulation (0–3 days post-implantation); **s2ON** — subscore in the second scanning session off medication and on stimulation (0—3 days post-implantation); **SD** — standard deviation.

## Acknowledgments

This work was supported by the Czech Ministry of Education: grant No. LH13256 (VES13-KontaktII), the Czech Ministry of Health: IGA MZ CR NT12282-5/2011, Charles University in Prague: research project PRVOUK-P26/LF1/4, grant No. PDF-IRG-1307 from the Parkinson's Disease Foundation (SH, KM & MLS), the International Max Planck Research School on Neuroscience of Communication: Function, Structure, and Plasticity (IMPRS NeuroCom; SH), LIFE – Leipzig Research Center for Civilization Diseases at the University of Leipzig – funded by the European Union, European Regional Development Fund and the Free State of Saxony within the framework of excellence initiative (SH & MLS), and the German Consortium for Frontotemporal Lobar Degeneration, funded by the German Federal Ministry of Education and Research (MLS). The authors thank the patients who participated in the study and Elizabeth Kelly for proofreading this manuscript.

## Figures and Tables

**Table 1:** Summary of demographic and clinical details of the 13 patients included in the study.

	ID	G	Age	DD	LD	s1OFF	s2OFF	s3OFF	s3ON	s4ON	CE	SE	DT	DF
	1	M	63	15	13	21	25	39	23	8	3	0.5	17	1
	2	M	53	11	7	45	32	42	19	17	0.5	1	3	1
	3	M	53	12	10	37	29	38	12	11	2	0.5	39	0
	4	M	45	14	6	47	28	45	16	10	1	0	59	1
	5	M	64	13	8	31	21	39	21	19	3	1	31	1
	6	M	53	12	9	43	24	43	10	11	4	3	3	1
	7	M	49	13	12	65	40	54	9	7	5	4	4	3
	8	M	55	12	9	46	24	31	16	7	4.5	3.5	38	1
	9	M	60	14	14	18	19	28	17	–	1	0	3	1
	10	F	42	9	6	33	27	28	11	13	2	1	10	1
	11	M	55	19	15	35	14	32	20	19	5	3	3	1
	12	M	43	9	7	34	35	40	20	22	1.5	1	17	1
	13	F	50	10	6	19	17	29	20	13	4.5	3.5	10	1
Mean	–	–	52.7	12.5	9.4	34.2	25.8	37.5	16.5	12.3	2.8	1.7	18.2	1.1
SD	–	–	7.0	2.7	3.2	9.9	7.3	7.7	4.6	4.2	1.7	1.5	18.0	0.6

Age is in years; CE — rating of cortical edema; DD — duration of the disease (years); DF — days after surgery to post-operative fMRI scan (days from surgery); DT — days between pre-implantation of fMRI scan and electrode implantation (days to surgery); G — gender (M — male/F — female); ID — patient's identification number; LD — duration of levodopa treatment (years); s1OFF — UPDRS-III score in the first scanning session off medication (pre-implantation); s2OFF — UPDRS-III score in the second scanning session off medication and off stimulation (0–3 days post-implantation); s3OFF — UPDRS-III score in the third measurement session off medication and off stimulation (~1 month post-implantation); s3ON — UPDRS-III score in the third session off medication and on stimulation (approx. 1 month post-implantation); s4ON — UPDRS-III score in the fourth measurement session off medication and on stimulation (approx. 1 year post-implantation); SE — rating of subcortical edema and SD — standard deviation.

**Table 2:** Summary of significant group EC differences related to penetration of DBS electrodes in the STN (*i.e.*, contrast = post-implantation − pre-implantation).

Structure	*P*_FDR‡_	*k*	*p*_unc_	*t*	*x*	*y*	*z*
Upper brainstem	**0.041**	**62**					
Maximum 1			2.7·10^−5^	6.10	−3	−28	−17
Maximum 2			0.002	3.45	−15	−31	−17
Lower brainstem (left)	**0.019**	**88**					
Maximum 1			8.1·10^−5^	5.39	−12	−37	−41
Maximum 2			5.1·10^−4^	4.31	−9	−43	−50
Maximum 3			0.002	3.52	−18	−43	−50
Lower brainstem (right)	**0.475**^a^	**21**					
Maximum 1			2.0·10^−4^	4.84	9	−34	−41
Maximum 2			4.5·10^−4^	4.38	15	−37	−50

Bold typeface letters denote obtained clusters and associated statistics at the cluster-level, while nested standard typeface letters are related to statistical values of the local maxima (at the voxel-level) identified within the respective cluster. DBS — deep-brain stimulation; EC — eigenvector centrality; *k* — number of significant voxels within the cluster; MNI — Montreal Neurological Institute; *p*_FDR‡_ — alpha value corrected for multiple tests at cluster-level using false discovery rate correction; *p*_unc_ — uncorrected alpha value; STN — subthalamic nucleus; *t* — peak *t*-statistic; [*x*, *y*, *z*] — coordinates of corresponding voxel in MNI-template anatomical space in millimeters;

a The right brainstem cluster did not survive the FDR cluster-level correction.
