# Supplementary material for: Resting-state functional magnetic resonance imaging of the subthalamic microlesion and stimulation effects in Parkinson's disease: Indications of a principal role of the brainstem
Source: Neuroimage Clin. 2015 Aug 21;9:264–74. doi: 10.1016/j.nicl.2015.08.008 (PMC4576412; doi:10.1016/j.nicl.2015.08.008)
Supplement: Supplementary Table S1 — UPDRS-III subscores of the 13 patients included in the study. ID — patient’s identification number; s1OFF — subscore in the first scanning session off medication (pre-implantation); s1ON — subscore in the first scanning session on medication (pre-implantation); s2OFF — subscore in the second scanning session off medication and off stimulation (0–3 days post-implantation); s2ON — subscore in the second scanning session off medication and on stimulation (0—3 days post-implantation); SD — standard deviation. [file mmc1.doc]

**Supplementary Table S1. UPDRS-III subscores of the 13 patients included in the study. ID – patient’s identification number; s1OFF – subscore in the first scanning session off medication (pre-implantation); s1ON – subscore in the first scanning session on medication (pre-implantation); s2OFF– subscore in the second scanning session off medication and off stimulation (0–3 days post-implantation); s2ON – subscore in the second scanning session off medication and on stimulation (0–3 days post-implantation); SD – standard deviation.**

|  | **Left Hemibody** | | | | **Right Hemibody** | | | | **Akinesia** | | | | **Rigidity** | | | | **Tremor** | | | | **Axial** | | | |
| --- | --- | --- | --- | --- | --- | --- | --- | --- | --- | --- | --- | --- | --- | --- | --- | --- | --- | --- | --- | --- | --- | --- | --- | --- |
|  | **s1** | | **s2** | | **s1** | | **s2** | | **s1** | | **s2** | | **s1** | | **s2** | | **s1** | | **s2** | | **s1** | | **s2** | |
| **ID** | **OFF** | **ON** | **OFF** | **ON** | **OFF** | **ON** | **OFF** | **ON** | **OFF** | **ON** | **OFF** | **ON** | **OFF** | **ON** | **OFF** | **ON** | **OFF** | **ON** | **OFF** | **ON** | **OFF** | **ON** | **OFF** | **ON** |
| 1 | 8 | 2 | 8 | 3 | 7 | 1 | 9 | 2 | 7 | 2 | 12 | 6 | 8 | 2 | 8 | 0 | 2 | 0 | 1 | 1 | 4 | 1 | 4 | 3 |
| 2 | 17 | 2 | 12 | 4 | 12 | 2 | 10 | 2 | 27 | 3 | 17 | 8 | 8 | 2 | 7 | 0 | 2 | 2 | 4 | 2 | 8 | 2 | 4 | 3 |
| 3 | 18 | 4 | 13 | 5 | 11 | 4 | 10 | 5 | 21 | 7 | 18 | 8 | 7 | 1 | 5 | 0 | 6 | 1 | 5 | 4 | 3 | 2 | 2 | 1 |
| 4 | 17 | 7 | 11 | 3 | 13 | 6 | 8 | 1 | 22 | 10 | 10 | 4 | 14 | 5 | 12 | 1 | 3 | 2 | 2 | 2 | 9 | 4 | 5 | 2 |
| 5 | 11 | 3 | 4 | 1 | 10 | 2 | 8 | 2 | 13 | 3 | 9 | 5 | 8 | 2 | 6 | 1 | 5 | 2 | 2 | 2 | 5 | 3 | 4 | 4 |
| 6 | 11 | 2 | 5 | 0 | 17 | 4 | 9 | 1 | 22 | 4 | 10 | 3 | 11 | 1 | 8 | 0 | 2 | 2 | 2 | 0 | 8 | 3 | 4 | 3 |
| 7 | 21 | 6 | 12 | 7 | 23 | 4 | 15 | 4 | 34 | 8 | 20 | 9 | 16 | 4 | 10 | 5 | 4 | 2 | 4 | 2 | 11 | 5 | 6 | 6 |
| 8 | 20 | 7 | 11 | 3 | 12 | 6 | 6 | 1 | 23 | 13 | 13 | 5 | 9 | 4 | 7 | 1 | 8 | 1 | 2 | 1 | 6 | 3 | 2 | 1 |
| 9 | 6 | 1 | 7 | 3 | 3 | 1 | 3 | 1 | 10 | 2 | 8 | 8 | 2 | 0 | 5 | 0 | 0 | 2 | 1 | 0 | 6 | 4 | 5 | 5 |
| 10 | 13 | 4 | 9 | 2 | 8 | 1 | 9 | 2 | 17 | 5 | 15 | 6 | 9 | 0 | 8 | 1 | 0 | 1 | 0 | 0 | 7 | 1 | 4 | 4 |
| 11 | 13 | 2 | 7 | 0 | 9 | 0 | 1 | 0 | 20 | 4 | 6 | 1 | 8 | 0 | 4 | 0 | 1 | 0 | 1 | 0 | 6 | 1 | 3 | 2 |
| 12 | 16 | 6 | 16 | 6 | 8 | 2 | 8 | 1 | 17 | 6 | 17 | 9 | 12 | 6 | 10 | 1 | 2 | 1 | 4 | 2 | 3 | 2 | 4 | 4 |
| 13 | 4 | 1 | 6 | 2 | 9 | 1 | 7 | 3 | 11 | 4 | 10 | 6 | 5 | 0 | 5 | 0 | 0 | 0 | 0 | 0 | 3 | 2 | 2 | 1 |
| **mean** | **13.5** | **3.6** | **9.3** | **3.0** | **10.9** | **2.6** | **7.9** | **1.9** | **18.8** | **5.5** | **12.7** | **6.0** | **9.0** | **2.1** | **7.3** | **0.8** | **2.7** | **1.2** | **2.2** | **1.2** | **6.1** | **2.5** | **3.8** | **3** |
| **SD** | **5.3** | **2.2** | **3.5** | **2.1** | **4.9** | **2.0** | **3.4** | **1.4** | **7.4** | **3.3** | **4.3** | **2.4** | **3.7** | **2.1** | **2.4** | **1.4** | **2.5** | **0.8** | **1.6** | **1.2** | **2.5** | **1.2** | **1.2** | **1.6** |
